# Improving the ‘tool box’ for robust industrial enzymes

**DOI:** 10.1007/s10295-017-1920-5

**Published:** 2017-04-11

**Authors:** J. A. Littlechild

**Affiliations:** Henry Wellcome Building for Biocatalysis, Biosciences, College of Life and Environmental Sciences, University of Exeter, Stocker Road, Exeter, EX4 4QD UK

**Keywords:** Thermophilic enzymes, Esterases, Lactonases, Epoxide hydrolases, Carbonic anhydrases

## Abstract

The speed of sequencing of microbial genomes and metagenomes is providing an ever increasing resource for the identification of new robust biocatalysts with industrial applications for many different aspects of industrial biotechnology. Using ‘natures catalysts’ provides a sustainable approach to chemical synthesis of fine chemicals, general chemicals such as surfactants and new consumer-based materials such as biodegradable plastics. This provides a sustainable and ‘green chemistry’ route to chemical synthesis which generates no toxic waste and is environmentally friendly. In addition, enzymes can play important roles in other applications such as carbon dioxide capture, breakdown of food and other waste streams to provide a route to the concept of a ‘circular economy’ where nothing is wasted. The use of improved bioinformatic approaches and the development of new rapid enzyme activity screening methodology can provide an endless resource for new robust industrial biocatalysts.This mini-review will discuss several recent case studies where industrial enzymes of ‘high priority’ have been identified and characterised. It will highlight specific hydrolase enzymes and recent case studies which have been carried out within our group in Exeter.

## Introduction

Many hydrolase enzymes including proteases, lipases, esterases and cellulases are already used commercially in industrial biocatalysis as documented in recent review chapters [56, 63, 66]. However, there is still an increasing need for the new enzymes to compliment the existing ‘Tool Box’ and to make these commercially available. The enzyme biocatalysts required for industrial processes need to withstand conditions that they are not usually exposed to within their natural environments such as high temperatures, extremes of pH, high salt, high substrate concentrations and the presence of organic solvents.

Extremophiles are organisms that have evolved to survive under many of these conditions and their intracellular and exported proteins often possess the desired attributes required for industrial applications.

This mini-review will concentrate on specific novel enzymes from characterised thermophilic archaeal and bacterial species isolated from marine and terrestrial ‘hot’ environments. In addition, enzymes sourced from metagenomes containing DNA and RNA from un-culturable microorganisms and their viruses will be included. Thermophilic enzymes from the archaea often offer additional novelty when compared to those from the thermophilic bacteria. The archaeal enzymes have evolved under different evolutionary pressure and generally represent more primitive enzymes. Different archaeal species have novel metabolic pathways that are not found in other microorganisms. For example, some species have modified versions of the Embden Meyerhof and Entner Doudoroff pathway involving a large number of novel enzymes [64] and have unusual pentose degradation pathways. Several archaeal enzymes are promiscuous regarding their activity to related substrates when compared to the equivalent enzymes isolated from bacteria or eukaryotes [13, 41, 57, 71]. This property to act on different structurally related substrates may be advantageous for some industrial processes.

The stability of an enzyme is dependent on maintenance of a functional structure which relies on a number of molecular interactions [12]. This property is also the case with a thermostable protein where the free energy of stabilisation is slightly higher than that of its mesophilic counterpart [32]. The overall stability of all proteins is marginal and they are easily denatured by a variety of changes in temperature and pH. The active form of a protein is held together using a combination of non-covalent forces including hydrogen bonds, ion pairs, hydrophobic bonds and Van der Waals interactions. When these interactions are disrupted, for example by elevated temperatures, both mesophilic and thermophilic proteins unfold into inactive but kinetically stable structures. Once unfolded in this manner the protein is prone to aggregation and chemical modification. Chemical modifications of the protein can also occur under other conditions and include cysteine oxidation, deamination of asparagine and glutamine residues and peptide bond hydrolysis. The unfolding of the protein when exposed to elevated temperatures may be reversible for smaller proteins, but is usually irreversible with larger proteins.

Some extremophilic microorganisms are able to grow at extremes of pH and are called acidophiles and alkalophiles, respectively. It is only the proteins exported from the cell that have to be stable under the pH conditions of the extreme growth environment. The proteins inside the cell do not have to withstand these extreme conditions as the intracellular pH is maintained around pH 5.0–6.0.

The use of more thermostable proteins, apart from being more cost effective with regard to enzyme use, can offer the option to run the process at elevated temperatures where non-natural substrates are more soluble. The temperature for operation of the industrial process needs to be balanced against the overall economics of the biocatalytic conversion. An example of a process which is commercially operated at 50 °C is the production of L-amino acids and L-amino acid analogues using a thermophilic archaeal L-aminoacylase enzyme that has been cloned and over-expressed from the archaeon *Thermococcus litoralis* [72]. This enzyme is used for the multi-ton commercial production of L-amino acids by Chirotech/Dow Pharma. More recently, the process operated by Chirotech/Dr Reddy’s [4] uses the addition of a racemase enzyme to convert the isomer not used by the enzyme to the isomer which is used, enabling a more efficient process with 100% conversion of a racemic substrate to one desired product [28].

What makes a protein more stable to elevated temperatures has been identified by studying the biochemical and structural features of a range of purified thermophilic proteins [45]. The structural features to increase thermostability as used by ‘nature’ include an increase in ionic interactions within the monomeric protein structure and at subunit interfaces for multi-subunit proteins. These offer most stabilisation when they occur in clusters as seen in many thermophilic enzymes including a thermophilic alcohol dehydrogenase enzyme from the hyperthermophilic archaeon *Aeropyrum pernix* which can be used industrially for chiral alcohol production [24]. The α-helices in the protein can be ‘capped’ by introduction of an acidic amino acid to neutralise the charge at the amino end of the helix and a basic amino acid to neutralise the charge at the acidic end of the helix. Many thermophilic proteins are also stabilised by hydrophobic interactions within the protein interior and at the subunit interfaces. This is often a feature of enzymes from thermophilic organisms such as the omega transaminase from *Sulfolobus solfataricus* [58] and pyroglutamyl carboxyl peptidase from *T. litoralis* [67]. The thermophilic proteins often have increased internal packing such as additional secondary structures and C-terminal extensions which can pack into the protein to fill unnecessary voids as seen in the glyceraldehyde 3-phosphate dehydrogenase from the thermophilic archaeon *S. solfataricus* [31]. Most thermophilic proteins have shorter surface loops and often the internal loops can be stabilised by metal ions [45]. An increased content of proline residues is seen in some thermophilic bacteria such as *Thermus* species which have a high G-C content in their DNA. Generally there is a reduction in amino acids that are unstable at high temperatures such as asparagines and cysteines except where they play an important catalytic role. Some hyperthermophilic aerobic archaea such as the *A. pernix* species use the introduction of a covalent disulfide bond into the protein to offer the necessary stability at high temperatures [24, 46].

## Esterase enzymes

The esterase enzymes are widely used industrial enzymes. They are able to carry out hydrolysis of an ester bond by a hydrolytic reaction using the classic ‘catalytic triad’ of three amino acids Ser, His, Glu or Asp [8]. They are relatively stable enzymes in organic solvents and have been widely used in both the hydrolytic and the reverse synthetic direction to carry out esterification and transesterification reactions. A general review on lipases was published by Bornscheuer [10]. Most lipolytic enzymes belong to the α/β hydrolase protein fold superfamily [53]. The catalytic serine residue in the α/β hydrolase fold esterases is usually located in a tight nucleophilic elbow with the consensus sequence Gly-X-Ser-X-Gly, although deviations from this consensus have been reported [9, 51].

However, esterase activity has also been reported for enzymes with a β lactamase fold [74] and an α/β/α hydrolase fold [73] or as a side activity for the zinc containing carbonic anhydrase enzymes [30]. The ESTHER database [42] divides the α/β hydrolase enzymes into over 140 families and superfamilies which are further assigned to groups C, H, L, and X.

Esterases are used for pharmaceutical intermediates due to the stereo-selectivity of their reactions, but also are finding many other uses as bulk enzymes in the healthcare, laundry and food industries and for degradation of domestic and agricultural biomass. There is always a demand for esterase enzymes with increased stability and selectivity for a variety of applications in industrial biotechnology. As part of a recent EU project ‘HOTZYME’ new esterase enzymes with different structural features and specificities have been identified and characterised. The first of these was found in the genome of a well characterised thermophilic archaeon, *Archaeoglobus fulgidus* [70]. The *Arch. fulgidus* is an anaerobic heterotrophic sulphate-reducing archaeon that grows at temperatures between 60 and 95 °C, with an optimal growth at 83 °C. It was isolated at Volcano Island in Italy 30 years ago. It was the first sulphur metabolising organism to have its genome sequence determined [40]. Some esterases and a lipase were earlier characterised from this organism including AFEST [47] which is a member of a hormone-sensitive lipase family and can be used for the biocatalytic conversion of poly δ-valerolactone being thermostable up to 90 °C. The first esterase Est-AF [16] and directed mutants of this enzyme have been designed for the industrial production of the (*S*)-enantiomer of ketoprofen that acts as an anti-inflammatory drug [37, 38].

**Fig. 1:**
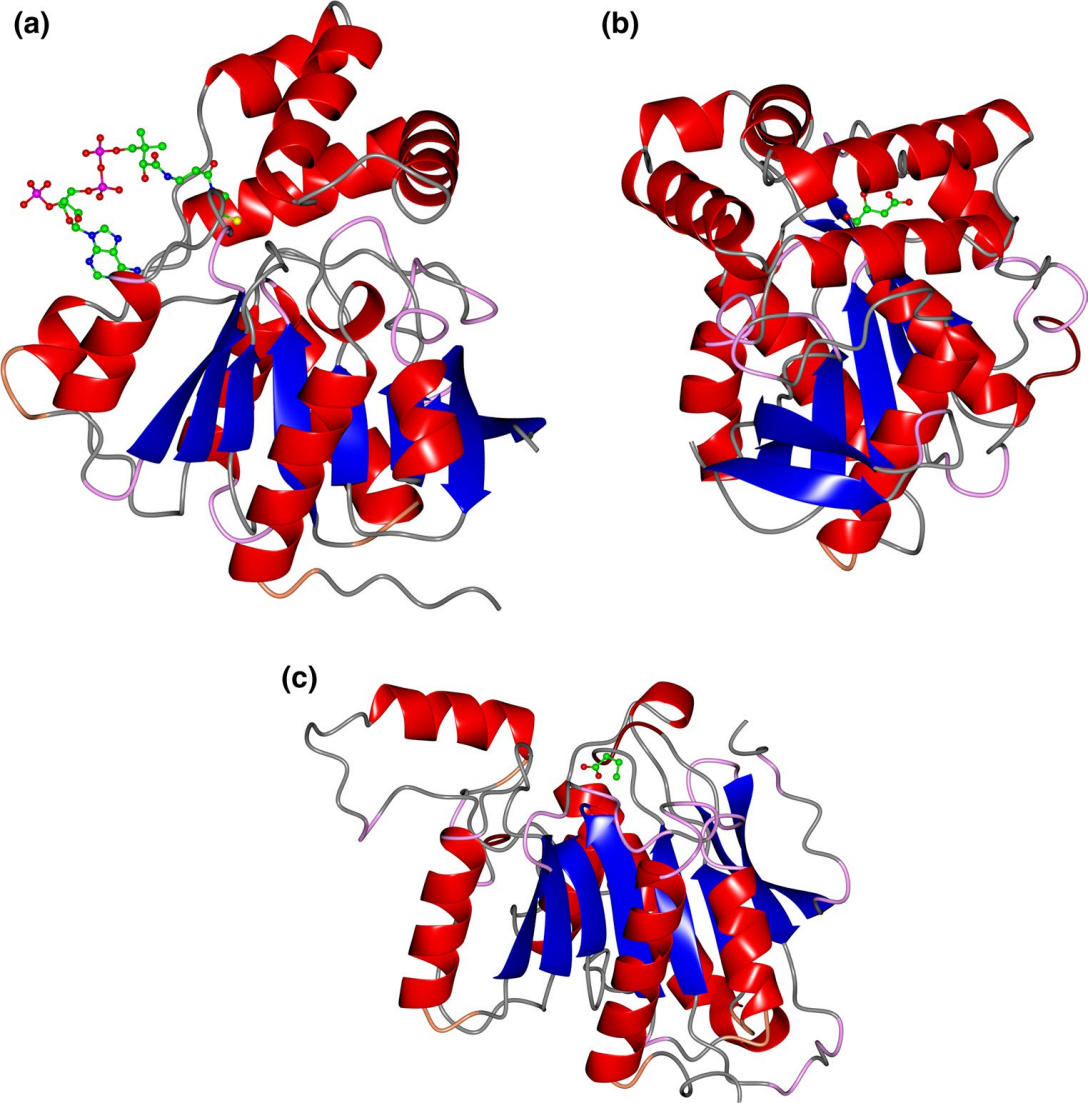
A cartoon representation of the crystal structures of the three thermophilic esterase enzymes described above. The helices are shown as *red cylinders* and the sheets as *blue arrows*. Other selected atoms are shown as *ball and stick* representation. **a** The *Archaeoglobus* archaeal esterase, AF-Est2, showing the larger core and smaller cap domains, with the bound CoA molecule and catalytic residues shown as a stick model PDB code 5FRD. **b** The *T. terrifontis* bacterial esterase, TtEst, showing D-malate which maps the alcohol-binding pocket of the active site PDB code 4UHE. **c** The second bacterial esterase from *T. terrifontis*, TtEst, showing the open active site and minimal cap domain. The substrate butyrate is bound shown in *ball and stick* representation which maps the carboxyl-binding pocket of the active site PDB code 5AOB. Images were generated using CCP4 MG [48]

The new carboxyl esterase, AF-Est2, belongs to the α/β hydrolase 6 family and the X group of the ESTHER classification. It has been cloned, over-expressed in *Escherichia coli* and biochemically and structurally characterised [61]. The AF-Est2 has very good solvent and pH stability and is very thermostable, showing no loss of activity after incubation for 30 min at 80 °C making it a good addition to the industrial esterase enzyme ‘tool box’. The structure of the esterase at high-resolution (PDB: 5FRD, Fig. 1a) revealed Coenzyme A (CoA) bound in the vicinity of the active site; however, this enzyme has no CoA thioesterase activity as described for related enzymes [5]. The pantetheine group of CoA partially obstructs the active site alcohol pocket of AF-Est2 suggesting that this ligand has a role in regulation of the enzyme activity. A comparison of the structure of AF-Est2 with the human carboxyl esterase 1 [5] which has CoA thioesterase activity, reveals that the cofactor is bound to different parts of the core domain in these two enzymes and approaches the active site from opposite directions.

Two other interesting thermophilic esterases have been characterised from a recently discovered thermophilic Planctomycetes species of bacterium named *Thermogutta terrifontis* [59, 60]. The *T. terrifontis* has been isolated from a terrestrial hot spring on Kunashir Island, Russia [68]. It is the first thermophilic member of the phylum Planctomycetes. This organism grows up to 70 °C, with an optimum growth temperature of 55–60 °C. Planctomycetes represents a deep distinct phylum in the bacterial domain and includes a large group of microorganisms with unique features not found in other bacteria such as reproduction by budding, presence of intra-cytoplasmic membranes which divide the cell into compartments, presence of a primitive nucleoid and the lack of peptidoglycan in the cell wall.

The first *T. terrifontis* esterase, TtEst, has been cloned and over-expressed in *E. coli* [59]. The enzyme is highly thermostable retaining 95% of its activity after incubation for 1 h at 80 °C. It has been biochemically characterised and shown to have activity towards small *p*-nitrophenyl *(p*NP*)* carboxylic esters with optimal activity for *p*NP-propionate. The structure of the enzyme has been determined without ligands bound in the active site (Fig. 1b) and in complex with a substrate analogue, D-malate and the product acetate (PDBs: 4UHC (native), 4UHD (acetate bound), 4UHE (malate bound), 4UHF (L37A mutant with butyrate bound)).

The bound ligands in the structure have allowed the identification of the carboxyl and alcohol-binding pockets in the enzyme active site. The results have also contributed to an understanding of substrate specificity and the subtle structural differences between esterase and lactonase enzymes. A comparison has been made of the alcohol-binding pocket in TtEst with the equivalent pocket in two other structurally related enzymes, a lactonase and a γ-lactamase. The 3-oxoadipate-enol lactonase from *Burkholderia xenovorans* (PcaD) (PDB code 2XUA) [3] has only 29% sequence identity to TtEst and the *Aureobacterium* species (−) γ-lactamase (Agl) (PDB code 1HKH) [44] with 30% sequence identity has shown that the catalytic triad residues and the position of the oxyanion hole are conserved between the enzymes. The PcaD and Agl show that the TtEst pocket that forms part of the active site has a much more polar and charged environment allowing the binding of organic acids such as D-malate where the distant carboxyl is coordinated by Arg139 and Tyr105. The PcaD and Agl enzymes have more hydrophobic substrate binding pockets, with the residues Arg139 and Tyr105 in TtEst replaced by Trp135 and Ile129 in PcaD and Trp204 and Leu125 in Agl. For PcaD and Agl, the active site is suited for the binding of structures such as lactone and γ-lactam rings. Similarly, the *Pseudomonas fluorescens* esterase with 30% sequence identity (PDB code 3HEA) [78] has an alcohol pocket which is lined with several hydrophobic phenylalanine side chains that should have affinity for the lactone ring. This would explain its lactonase activity towards caprolactone [11] in addition to its esterase activity. This enzyme and the Agl enzyme both have perhydrolase activity. They are able to produce hypochlorous and hyperbromous acid in a side reaction at acidic pH [6] and were originally named as non-cofactor chloroperoxidases [27].

Mutant enzymes have been constructed to extend the substrate range of the TtEst esterase to accept the larger butyrate and valerate *p*NP-esters. The crystal structure of the Leu37Ala mutant shows the butyrate product bound in the carboxyl pocket of the active site. The structure shows an expansion of the pocket that binds the substrate carboxyl group which allows the observed activity towards *p*NP-butyrate [59].

The second *T. terrifontis* esterase, TtEst2, shows substantial differences when compared with the TtEst1. A large difference is observed with the absence of the usual ‘cap’ domain found in most other esterase enzymes which results in an open substrate binding site that is solvent accessible (Fig. 1c) [60]. This TtEst2 enzyme belongs to the α/β-hydrolase family 3 in the Pfam classification [21]. It has been characterised biochemically and shown to have activity towards small *p*-nitrophenyl (*p*NP) carboxylic esters with optimal activity for *p*NP-acetate. The enzyme is not as thermostable as TtEst1, but still retains 75% of its activity after incubation for 30 min at 70 °C. The structure of this enzyme has been determined in the native form and in complex with the carboxylic acid products propionate, butyrate and valerate. The PDB codes 5AO9 (native), 5AOa (propionate bound), 5AOb (butyrate bound), 5AOc (valerate bound) have been deposited. The structures with bound ligands have allowed the identification of the carboxyl-binding pocket in the enzyme active site. Since this enzyme has a very open active site compared to other esterase enzymes it has the potential to be active towards larger substrates.

## Lactonases

The specific cleavage of a lactone ring is an important activity of interest to many pharmaceutical companies. Lactones have been shown to be important communication molecules between microbial cells and the topic has been reviewed [75].

The lactonase enzymes identified to date fall into three structurally diverse groups: the enol lactonases, the gluconolactonases and the quorum-sensing lactonases. Phosphotriesterase-like lactonases were identified in the archaeal species *S. solfataricus* and *S. acidocaldarius* [1, 49, 50, 54]. These enzymes catalyse the hydrolytic cleavage of the intramolecular ester bond in lactones and acyl-homoserine lactones to give the corresponding hydroxyacylic acids. This class of lactonases also have a promiscuous phosphotriesterase activity towards organophosphate compounds and have the potential to be used for remediation of contaminated soils. However, the natural role for these lactonases is to break down lactones that are thought to play a role in quorum sensing between microorganisms and are involved in biofilm formation [14, 39] and also in the expression of virulence factors that are of interest in medicinal and biotechnological applications [25]. Therefore, the enzymatic degradation of lactones could be used to interrupt quorum-sensing signalling pathways and to control microbial communication so that they cannot form communities.

A lactonase of this class has been identified, cloned, over-expressed and characterised [34] from *Vulcanisaeta moutnovskia* a hyperthermoacidophilic crenarchaeon isolated from a solfataric field in Kamchatka (Russia) [23, 55]. This lactonase was studied with view to its substrate specificity for biocatalytic applications of interest to the pharmaceutical industries. The lactonase converted lactones and acyl-homoserine lactones with comparable activities. A promiscuous, lower activity was observed with organophosphates and minor activity was observed with carboxyl esters. The catalytic activity was strictly dependant on bivalent cations (Cd^2+^ > Ni^2+^ > Co^2+^ > Mn^2+^ > Zn^2+^) and was most active at pH 8.0, and at 80 °C. The enzyme has high activity towards linear γ-lactones with hydrophobic side chains of variable lengths from γ-butyrolactone (no side chain) and γ-valerolactone which has a methyl side chain and γ-dodecalactone which had a seven-carbon side chain. It was shown that the enzyme has activity to whiskey lactone and δ-dodecalactone. No measurable activity was seen for mevalonolactone or δ-decalactone. The lactonase enzyme had increased activity towards the D form of these substrates [34].

The recent structure of the *V. moutnovskia* lactonase has been carried out in complex with a long chain fatty acid which maps the position of the substrate binding pocket [26]. This enzyme belongs to the amidohydrolase enzyme superfamily which has a (β/α)_8_-barrel structural fold. The two bound cobalt ions in this enzyme that are essential for activity are located in at the C-terminus of the β barrel in the crystal structure. In the proposed catalytic cycle, the metal ions activate a bridging water molecule through proton abstraction. The resulting hydroxide ion then performs a nucleophilic attack on the C_1_ of the lactone ring resulting in hydrolysis [26].

The high thermal stability of this class of lactonase enzymes as well as their broad substrate specificity for different lactones makes them interesting new enzyme for the biocatalytic ‘tool box’.

The enol lactonases are members of the α/β hydrolase superfamily and are closely related to the esterases. The 3-oxoadipate-enol lactonase from *B. xenovorans* (PcaD) (PDB code 2XUA) [7] has been mentioned above. A thermophilic member of this family has been identified, cloned and studied from the archaeon *Carboxydothermus hydrogenoformans* [62].

## Epoxide hydrolases from extremophilic metagenomes

An important enzyme activity of interest to the pharmaceutical industry is the ability to catalyse the hydrolysis of an epoxide ring by addition of a molecule of water to form a vicinal diol as a product [36, 76]. Enzymes found in nature that can carry out this reaction play an important role in the detoxification of reactive xenobiotics or endogenous metabolites and in the formation of biologically active mediators. The so-called ‘epoxide hydrolases’ are already used for the production of optically pure epoxides and diols which are important synthons for the preparation of fine chemicals and drugs, for example the chiral precursors of β-blockers [35, 52]. These enzymes are found in two classes which have different structure and mechanisms. The most studied class has the α/β hydrolase fold which is characteristic of the esterase enzymes described above. The other less studied class is called the limonene epoxide hydrolases (LEHs) after the substrate, limonene, that they were shown to hydrolyse. The LEH enzyme active site contains three residues (Asp, Arg, and Asp) that have been proposed to act in a concerted fashion to activate a water molecule which is able to open the epoxide ring without the formation of a covalently bound alkyl-enzyme intermediate [2, 29]. A recent review has described the importance and industrial interest of these hydrolase enzymes [77].

**Fig. 2:**
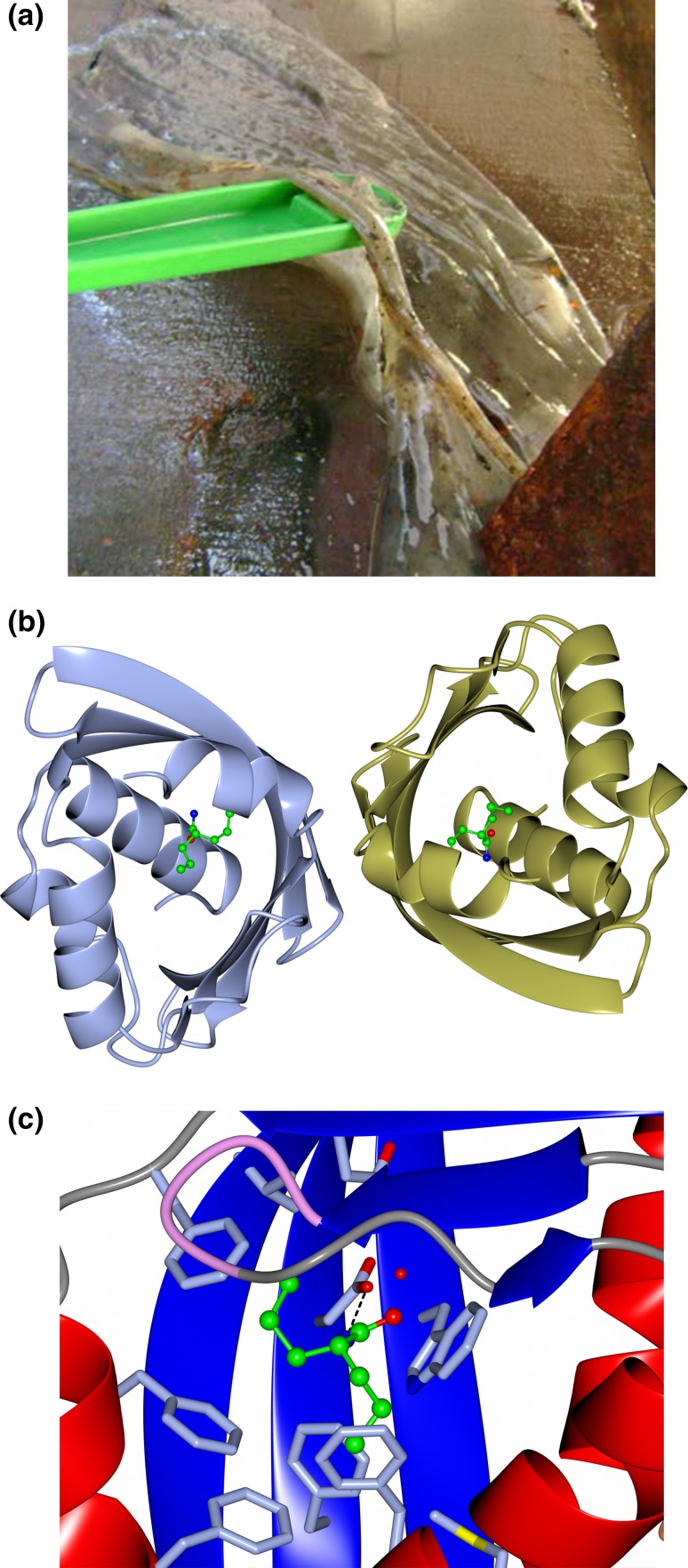
**a** The microbiological mat at the Tomsk sampling site, Parabel, Tomsk Region, West Siberia, Russia, which provided the metagenome DNA sample where the LEH was identified. Picture kindly provided by Prof. Elizaveta Bonch-Osmolovskaya. **b** A cartoon representation of the dimeric Tomsk-LEH structure in complex with an inhibitor valpromide shown in *ball and stick* representation which is bound at the active site. PDB code 5IG. **c** A *close-up* representation of the active site of Tomsk-LEH with the active site residues and inhibitor highlighted. The *red sphere* represents the active site water molecule. Images were generated using CCP4 MG [48]

Recently, as part of a thermophilic metagenomic project two new thermostable epoxide hydrolases of the limonene class have been discovered. The metagenomes were isolated in Russia and China from hot terrestrial environments growing at 46 and 55 °C, respectively, and at neutral pH. The microbial mat which was sampled at the Russian Tomsk site is shown in Fig. 2a. A bioinformatic approach was used to identify the genes coding for these industrially important enzymes using the mesophilic *Rhodococcus* LEH as a search. The two new thermophilic LEHs have been cloned and over-expressed in *E. coli* and the resultant proteins characterised both biochemically and structurally [19]. The atomic coordinates and structure factors of the crystal structures obtained have been deposited in the PDB: 5AIF (Tomsk-LEH native structure), 5AIG (Tomsk-LEH valpromide complex), 5AIH (CH55-LEH native structure) and 5AII (CH55-LEH PEG complex). The overall structure of the Tomsk-LEH from the Russian-sourced metagenome is shown in Fig. 2b and the active site showing the catalytic water is shown in Fig. 2c. The new LEH enzymes have attracted industrial interest since they are more thermostable than the available LEH and have different stereo-preference for the different isomers of limonene-1,2-epoxide. They have already been used in pilot-scale biotransformations for efficient epoxide hydrolase-catalysed resolutions of (+)- and (−)-*cis*/*trans*-limonene oxides which have important industrial applications [20].

## Carbonic anhydrases

Carbonic anhydrase enzymes (CAs) catalyse the reversible hydration of carbon dioxide to bicarbonate. The bovine and human enzymes of the α-carbonic anhydrase class have been widely studied and are known to be very efficient catalysts of CO_2_ hydration. Most of these enzymes also have a side activity as an esterase although the mechanism involves a zinc ion and not the catalytic triad found in most esterases. The carbonic anhydrase reaction mechanism involves an attack of a zinc-bound hydroxide ion onto a CO_2_ molecule which is bound in a hydrophobic pocket of the enzyme. The resulting zinc-coordinated bicarbonate ion is removed from the metal ion by water. There is then a rate-limiting step where an intramolecular proton is transferred from the zinc-bound water molecule to a histidine amino which serves as a proton shuttle between the metal centre and buffer molecules in the reaction medium [43].

Carbonic anhydrase has industrial applications as a catalyst for CO_2_ capture from the environment and from industrial waste streams. However, the biocatalyst used has to be stable to the temperature and other harsh conditions that it is exposed to during the process.

**Fig. 3:**
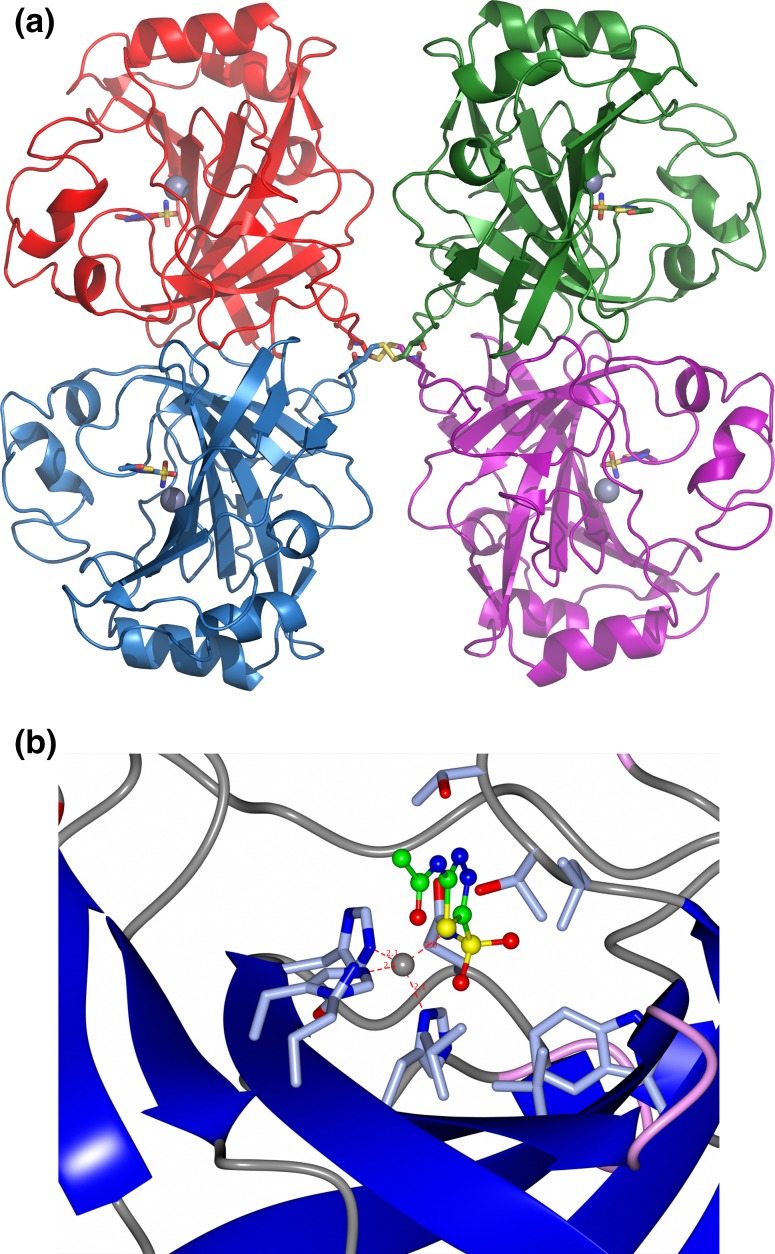
**a** A cartoon representation of the tetramer structure of the α-carbonic anhydrase from *T. ammonificans* showing each subunit in a different colour. The active site zinc is shown as a *sphere* in each subunit together with an inhibitor in *ball and stick* representation bound at each active site. The disulfide bonds at the centre of the tetramer which add stability to the enzyme are shown in *yellow*. **b** A *close-up* representation of the active site of the enzyme showing the catalytic zinc molecule and the inhibitor acetazolamide bound to the active site. *Side chain* residues co-ordinating the zinc ion and residues making up the active site are highlighted. PDB code 4COQ. Images were generated using CCP4 MG [48] and PyMOL [15]

The thermophilic bacteria appear to contain high activity α-carbonic anhydrase enzymes which are related to the bovine and human carbonic anhydrase enzymes, whereas the archaea have carbonic anhydrases that have different structures and mechanisms. There are six distinct families of CAs (α, β, γ, δ, ζ, and η) [22, 69]. The amino acid sequences are conserved between each family; however, there is no sequence or structural similarity between the different families. The CA activity always requires the presence of a catalytic zinc ion which is coordinated to either histidine or cysteine amino acids depending on the class of the enzyme [65]. A stable robust α-carbonic anhydrase has been identified in the thermophilic bacterium *Thermovibrio ammonificans.* The enzyme has been cloned and over-expressed in *E. coli*. This protein has been characterised both biochemically and structurally [33]. The crystal structure of this enzyme has been determined in its native form and in two complexes with bound inhibitors. The overall structure of the enzyme is unique since it forms a tetrameric structure rather than the dimer reported for related enzymes. The *Thermovibrio* carbonic anhydrase is stabilised by a unique core in the centre of the tetramer which is formed by two intersubunit disulfide bonds and a single lysine residue from each monomer (Fig. 3a). The structure of this central core region protects the intersubunit disulfide bonds from reduction. The catalytic zinc ion coordinated to histidine residues that is observed in the active site of the *Thermovibrio* enzyme is shown in Fig. 3c.

Another thermophilic bacterial α-carbonic anhydrase has been described from *Sulfurihydrogenibium yellowstonense.* This carbonic anhydrase is also thermostable and is a dimer stabilised by ionic networks [17]. A review of the different types of thermophilic carbonic anhydrase enzymes has recently been published [18].

## Summary

This mini-review has provided a selection of examples demonstrating the use of ‘Natures Catalysts’ to provide a ‘tool box’ of biocatalysts for sustainable applications in industrial biotechnology. This has been highlighted using different thermophilic hydrolase enzymes identified in thermophilic genomes and metagenomes from both bacterial and archaeal sources. These enzymes are robust to the conditions required for their different industrial applications. It is expected that the number of these enzymes used commercially will increase due to their inherent stability and novel specificities. The enzymes can be cloned and over-expressed in easily growing hosts such as *E. coli* which can provide sufficient quantities of the purified enzymes for different biocatalytic applications. For larger scale industrial applications, where the enzyme is required in kg quantities, an alternative fungal host system that exports the enzyme into the growth media could be developed.

The stability of the biocatalyst is an important issue since to be economically viable the enzyme needs to be reused for several biocatalytic cycles. Immobilisation of the enzyme can often increase its stability and will also allow it to be easily recovered for reuse. The cost of the enzyme biocatalyst is usually the most expensive component of the industrial biotransformation and this must be matched to the value of the end product or process.

The use of enzymes is expected to grow in the next year with bio-based materials, chemicals and sustainable processes predicted to rise globally to over 7.4 million metric tons in 2018 (Lux Research analysts). The time required to source the best enzyme catalyst and its optimisation for the desired process, is still a limiting factor for a cost-effective biocatalytic route. There is, however, a large natural resource available to search for novel enzymes to add to the currently available ‘tool box’.

The adoption of new commercial biocatalytic processes will be important to achieve the goal of a sustainable ‘circular economy’ and to address important global challenges.
